# From Taboo to Touchscreen: A Qualitative Study of Digital Sexual and Reproductive Health Intervention for Bangladeshi Adolescents

**DOI:** 10.2196/78514

**Published:** 2025-10-07

**Authors:** Kamrul Hasan, Hassan Rushekh Mahmood, Saraban Tahura Ether, Tanvir Hayder, Soaiba Zannat, Abu Sayeed, A M Rumayan Hasan, Ahmed Ehsanur Rahman, Chungyi Chiu, Anisuddin Ahmed, Md Refat Uz Zaman Sajib

**Affiliations:** 1 Maternal and Child Health Division (MCHD), icddr,b, Dhaka, Bangladesh; 2 Department of Health Systems and Populations, Faculty of Medicine, Health and Human Sciences, Macquarie University, Sydney, Australia; 3 Health System and Population Studies Division (HSPSD), icddr,b, Dhaka, Bangladesh; 4 Department of Health and Kinesiology, University of Illinois Urbana-Champaign, Champaign, IL, United States; 5 Global Health and Migration Unit, Department of Women's and Children's Health, Uppsala University, Akademiska sjukhuset, Uppsala, 751 85, Sweden, 46 0005617861

**Keywords:** adolescent health, sexual and reproductive health, mHealth, health education, communication, bangladesh, SRH, mobile health

## Abstract

**Background:**

Adolescents in Bangladesh, approximately one-fifth of the total population, face significant challenges accessing relevant sexual and reproductive health (SRH) information, with the added burdens of cultural taboo, limited accessibility, and poor communication channels. Traditional adolescent-friendly approaches have shown limited effectiveness in addressing these challenges. In response, Mukhorito was developed as a peer-led, mobile-based digital platform to facilitate SRH, education, and communication among ninth-grade students.

**Objective:**

This study explored the feasibility and constraints of piloting the Mukhorito app to enhance adolescent SRH education in Bangladesh. It also sought to determine the self-reported usage, usability, and effect on knowledge and peer communication of the app, as well as to identify implementation and adoption challenges.

**Methods:**

A qualitative design was applied in the context of a broader mixed-methods study. Data were collected through 6 in-depth interviews, 3 key informant interviews, and 1 focus group discussion from 19 participants, including students, peer leaders, teachers, and government representatives, across 3 secondary schools (a boys’ school, girls’ school, and coeducational school) in the Feni district. Thematic analysis was conducted using NVivo software (QSR International) under Braun and Clarke’s guidelines.

**Results:**

The Mukhorito app was perceived as a valuable tool to meet the SRH needs of adolescents in Bangladesh owing to its well-structured, easily navigable content with relatable stories. Participants described increased awareness of critical SRH issues, including reproductive health, nutrition, early marriage, violence against adolescents, and drug addiction, and reported sharing this knowledge with their families and communities. The app was seen as creating a safe space to discuss previously taboo SRH topics, reducing cultural reluctance to communicate, and promoting peer-to-peer communication. Interactive sessions were noted to strengthen decision-making skills and leadership qualities. Many users reported feeling more comfortable discussing SRH issues without shyness. However, challenges such as limited smartphone access, unreliable internet connection, and economic factors hindered adoption, especially in rural settings. Participants suggested the integration of the Mukhorito app in school curricula, aligning it with the government adolescent health program, adding visually rich and interactive content (eg, short dramas, videos, and animations), and enabling offline access to address connectivity challenges and maximize the effectiveness of the app.

**Conclusions:**

Mukhorito possesses strong potential as a culturally relevant, digital SRH education tool for Bangladeshi adolescents. The app enabled knowledge and openness in SRH discourse. Alignment with national health programs and enhanced app functionality may promote greater and more sustainable adolescent health.

## Introduction

Adolescence marks a pivotal stage of growth involving rapid physical, emotional, and social development, underscoring the importance of prioritizing investments in adolescent health and well-being [1]. Globally, the adolescent population is estimated at 1.2‐1.3 billion aged 10‐19 years, making up approximately one-sixth of the world’s population [2-4]. The number is rapidly increasing and is projected to grow further in the coming decades, particularly in low- and middle-income countries (LMICs), including Bangladesh, where around 90% of the world’s adolescents live [5,6].

Adolescents encounter considerable obstacles in accessing sexual and reproductive health (SRH) information and services worldwide, particularly in LMICs [7]. These challenges often involve limited awareness, a lack of youth-focused services, and socio-cultural constraints [8]. Geographic distance, costs, and privacy issues further limit adolescents’ access to SRH services [9]. In addition, cultural stigmas, minimal parental communication, and resource shortages further hinder young people’s access to SRH information [10]. To improve adolescent health outcomes, prioritizing SRH rights and services for adolescents is crucial [7].

In Bangladesh, adolescents constitute nearly one-fifth of the population, accounting for approximately 36 million individuals [11-13]. Despite their large numbers, this demographic faces significant health-related challenges, including access to crucial SRH information, counseling, and services concerning menstruation, reproductive tract infections, family planning, and more [14,15]. Furthermore, cultural taboos and a lack of supportive environments in educational settings perpetuate stigmatization around menstruation, reproductive health, and other SRH-related topics [11,14], resulting in high rates of child marriage, early childbearing, and unintended pregnancies among adolescents [13].

Recognizing the distinctive needs of adolescents, the Government of Bangladesh (GoB) launched a national strategy for adolescent health in 2017. This initiative emphasizes the importance of adolescent-friendly health services, as well as SRH education and resources, and introduces school-based health programs [11]. Under this program, GoB trained peer educators and teachers to deliver SRH education and increase awareness at the secondary school level [16]. With the rise of digital learning and mobile health solutions, GoB encourages the use of innovative, accessible health care delivery models, creating opportunities to integrate digital tools into adolescent health promotion. In Bangladesh, adolescents are increasingly accessing mobile devices, and while phone use is often restricted during classroom hours, there are no national age-based prohibitions on accessing public digital health resources. Recent mobile health (mHealth) initiatives, led by both government and nongovernmental organizations (NGOs), have piloted educational apps and SMS text messaging–based learning for adolescents [17,18]. These experiences highlight the feasibility of incorporating mobile app–based solutions to complement existing government school–based programs by strengthening peer communication and access to reproductive health information, services, and care [19].

Previous mHealth initiatives in Bangladesh, such as the GoB-United Nations Children’s Fund (UNICEF) “Adolescent Health” app, have sought to expand adolescents’ access to health information and public services [18]. This platform delivers essential SRH, nutrition, and hygiene content alongside service directories and referral options, improving access to static resources. However, its design is primarily one-way in nature, lacking interactive, participatory elements that foster dialogue, peer-to-peer learning, or leadership development. Similarly, other mHealth education interventions based on phone calls and SMS text messaging have demonstrated value in improving menstrual hygiene knowledge [17]; however, broader evidence from the literature indicates that many still face challenges in integrating user perspectives and culturally sensitive strategies for addressing taboo SRH topics and sustaining user engagement.

Thus, acknowledging the demand for innovative solutions to enhance adolescent SRH outcomes, we proposed a mobile app named Mukhorito (meaning “joyful” in Bengali and pronounced as “Moo-kho-ree-to” or ˈmuː.kʰo.ɾi.to) for leveraging peer communication, fostering an interactive and supportive learning environment, and providing tools for knowledge retention to improve the effectiveness of such GoB initiatives. This app creates secure digital spaces for peer interactions and distance learning through age-specific, structured, and interactive modules, which expand beyond the static offerings of current offline or digital resources [20]. Moreover, Mukhorito incorporates mechanisms to track participation in school-based adolescent health programs, collect feedback, and implement strategies to retain knowledge, with a specific focus on improving adolescent SRH education and outcomes.

This Mukhorito app was assessed using a mixed-methods study design. In this paper, qualitative methods are used to explore the opportunities and challenges of using the digital health platform for adolescent SRH services in Bangladesh. Our exploration focused on factors, such as how mobile app-based platforms can harness peer communication, identify potential barriers to adoption, and their impact on enhancing SRH knowledge, as well as strategies for developing scalable, culturally sensitive digital health interventions. By examining these factors, this study will contribute to enhancing user experience and the effectiveness of digital tools in advancing adolescent health education and suggest pathways forward for sustainable, impactful SRH initiatives in Bangladesh and other LMICs with similar contexts.

## Methods

### Mukhorito App Intervention

The GoB initiated a school-based adolescent health program countrywide, which trained over 5000 peer educators (among ninth-grade male and female students) and 9000 teachers from 21 districts and developed a manual on SRH regarding health concerns for ninth-grade students and their teachers [16]. These trained teachers and students were responsible for disseminating information and raising awareness about SRH among their peers. To complement ongoing SRH education by the GoB under the school-based adolescent health program, Mukhorito aimed to leverage the promise of growing digital literacy and widespread smartphone access (>90%) among adolescents in Bangladesh [21].

**Figure 1.**
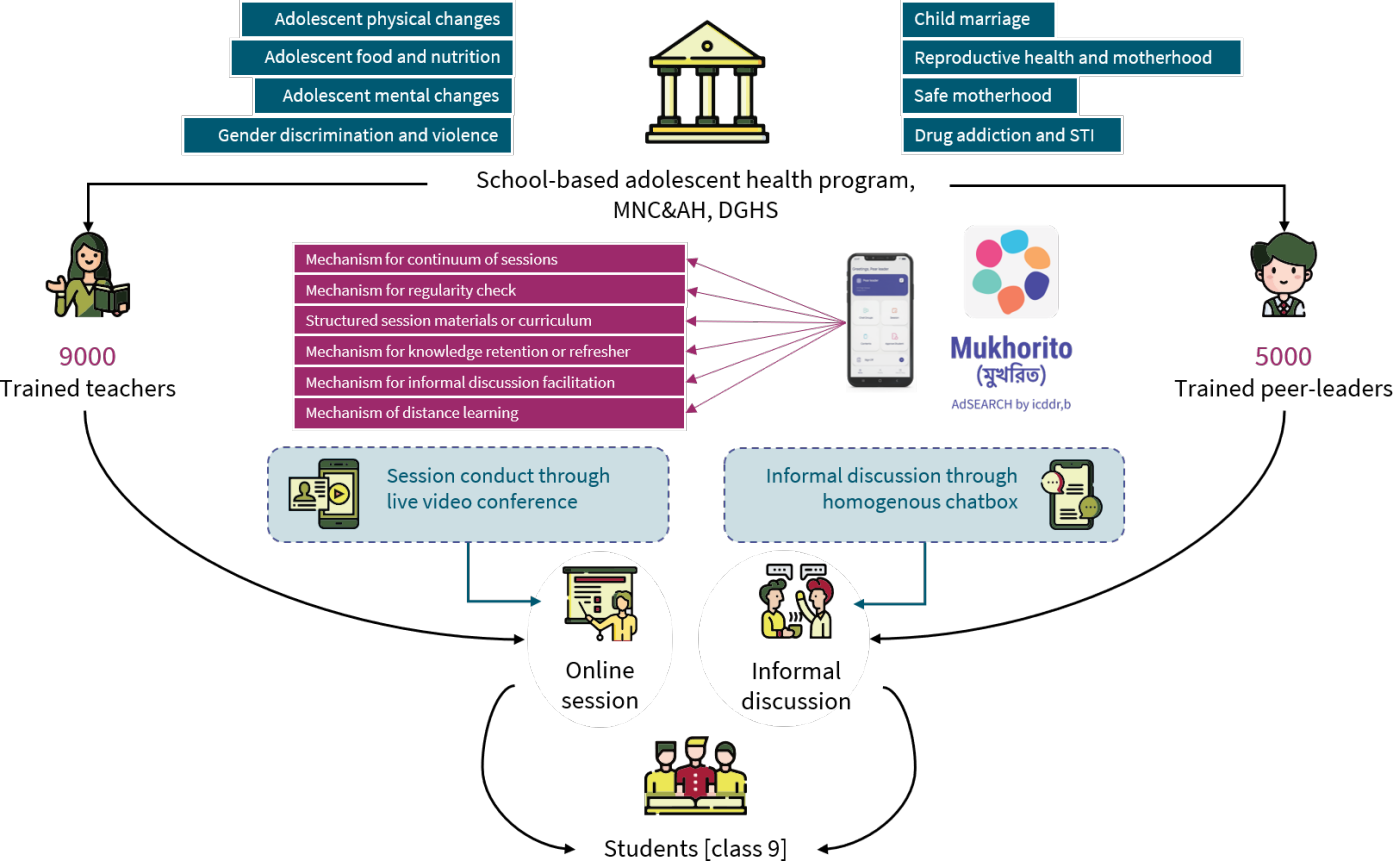
Conceptual framework of the Mukhorito study. MNC&AH: maternal, newborn, child, and adolescent health, DGHS: directorate general of health services; STI: sexually transmitted infection.

Mukhorito is an Android-based mobile app, as most people in our country context use Android mobiles, designed to facilitate safe and comfortable peer interaction and provide a structured learning environment for addressing SRH issues among adolescents. Mukhorito provided age-specific, GoB-accredited, self-paced, and interactive facilitated learning modules in eight domains: (1) adolescent physical changes; (2) adolescent food and nutrition; (3) adolescent mental changes; (4) gender discrimination and violence; (5) child marriage; (6) reproductive health and motherhood; (7) safe motherhood; and (8) drug addiction and sexually transmitted infections. Each domain integrated flashcards and posters, enhancing knowledge retention and supporting ease of learning. This multimodal content enabled students to engage with these materials at their convenience (Figure 1). Moreover, peer leaders facilitated 8 online sessions aligned with these domains, fostering group discussion and interactive learning. Furthermore, built-in quizzes assessed participants’ understanding and learning progress connected with class leaderboards. Mukhorito also included a school-based chat room where students could discuss SRH-related concerns anonymously with their peers and peer leaders. This unique feature provided a safe space for discussion. They also got access and connected to the national helpline, health facilities, and law enforcement support from the app. With all these interactive learning and engagement features, Mukhorito aimed to be a comprehensive, sustainable digital solution for promoting SRH awareness, service uptake, and support in the secondary school setting in Bangladesh.

### Study Design and Site

This study was a part of a broader implementation research (Experimental nonrandomized before-after design), mixed methods designed to understand the app’s usability, acceptability, level of adoption, and utility for peer communication and digital capacity-building facilitation on adolescent sexual and reproductive health and rights issues. For this paper, we focused on qualitative research methods, including in-depth interviews (IDIs), focus group discussion (FGD), and key informant interviews (KIIs), to explore the opportunities and challenges to the use of Mukhorito. The study was conducted in 3 secondary schools—a boys’ school, a girls’ school, and a coeducational school—located in the Feni district of Bangladesh. These schools were selected by consulting with the directorate of secondary and higher education (DSHE) and the directorate general of health services (DGHS) as the GoB school–based adolescent health program has already been run in these schools and equipped with trained peer leaders and teachers to create awareness about adolescent health.

### Study Participants and Sample

The study used purposive sampling to select respondents who were users or well-informed about the intervention. The researchers communicated with the students and peer leaders face-to-face at their school, while the researcher communicated over the phone with teachers and GoB Program Officials to schedule the interview time at their convenience. A total of 19 participants were included for 6 IDIs, 3 KIIs, and an FGD: 13 were ninth-grade students, including 3 general students and 10 peer leaders, 3 teachers, and 3 GoB Program Officials. The 3 IDIs were conducted with students who had used the app, each from a different school, and 3 IDIs were conducted with teachers who were from 3 different schools to gather comprehensive insights on the app usage. The teachers got the government training and were responsible for supervising the students and peer leaders to solve any problem, such as guiding on how they would conduct online sessions and answering any questions from students and peer leaders. The FGD with 10 peer leaders across 3 schools was conducted to gather intensive information in a supportive environment. To capture broader implementation perspectives, 3 KIIs included one participant from the District Education Office (DEO) in Feni under the DSHE and 2 from the DGHS, all of whom were involved in school-based adolescent health programs and worked with this study collaboratively. This diverse respondent group provided a comprehensive understanding of the app’s feasibility and its impact on adolescent SRH service access and usage.

### Data Collection

Data collection took place from March to June 2024, following the launch and introductory workshops of the app. The data were collected by anthropologists experienced in qualitative methods. All interviews and the FGD were facilitated by the first author (KH), and field notes were taken by a research assistant. To ensure that the data gathered was pertinent to each participant’s role, distinct guides were developed for students, teachers, peer leaders, and GoB program officials. The guides were internally tested and piloted, resulting in some changes to improve flow. This tailored approach allowed for a more nuanced understanding of each group’s experience and perspective regarding the digital platform of adolescent health learning. Before starting the interview, the researchers built up a rapport through general conversation and explained the aim of this research. The interviews with students, peer leaders, and teachers were conducted in the schools, while interviews with stakeholders took place in their offices at their convenience. All interviews were held in a private and quiet room to ensure confidentiality. This setting provided a comfortable environment where participants could freely and effectively share their experiences. For the FGD, homogeneity was ensured within the group for comfortable discussion; every participant was a ninth-grade student and a peer leader. The average length of the interviews was 25 minutes for IDIs, 27 minutes for KIIs, and the FGD took 57 minutes. During the interviews, field notes were taken to capture the context, the interaction between the researcher and the respondent, and the initial thoughts on the interview contents. These notes were expanded at the end of each day to capture any additional observations or reflections. This iterative process helped to identify information gaps or newly emerging insights, ensuring a more comprehensive and detailed understanding of the study’s findings. Saturation was considered reached when additional interviews no longer provided new insights relevant to the study’s objectives. This was determined through iterative transcript reviews, where themes began to repeat without the emergence of novel ideas, indicating code and meaning saturation [22].

### Data Management and Analysis

**Figure 2.**
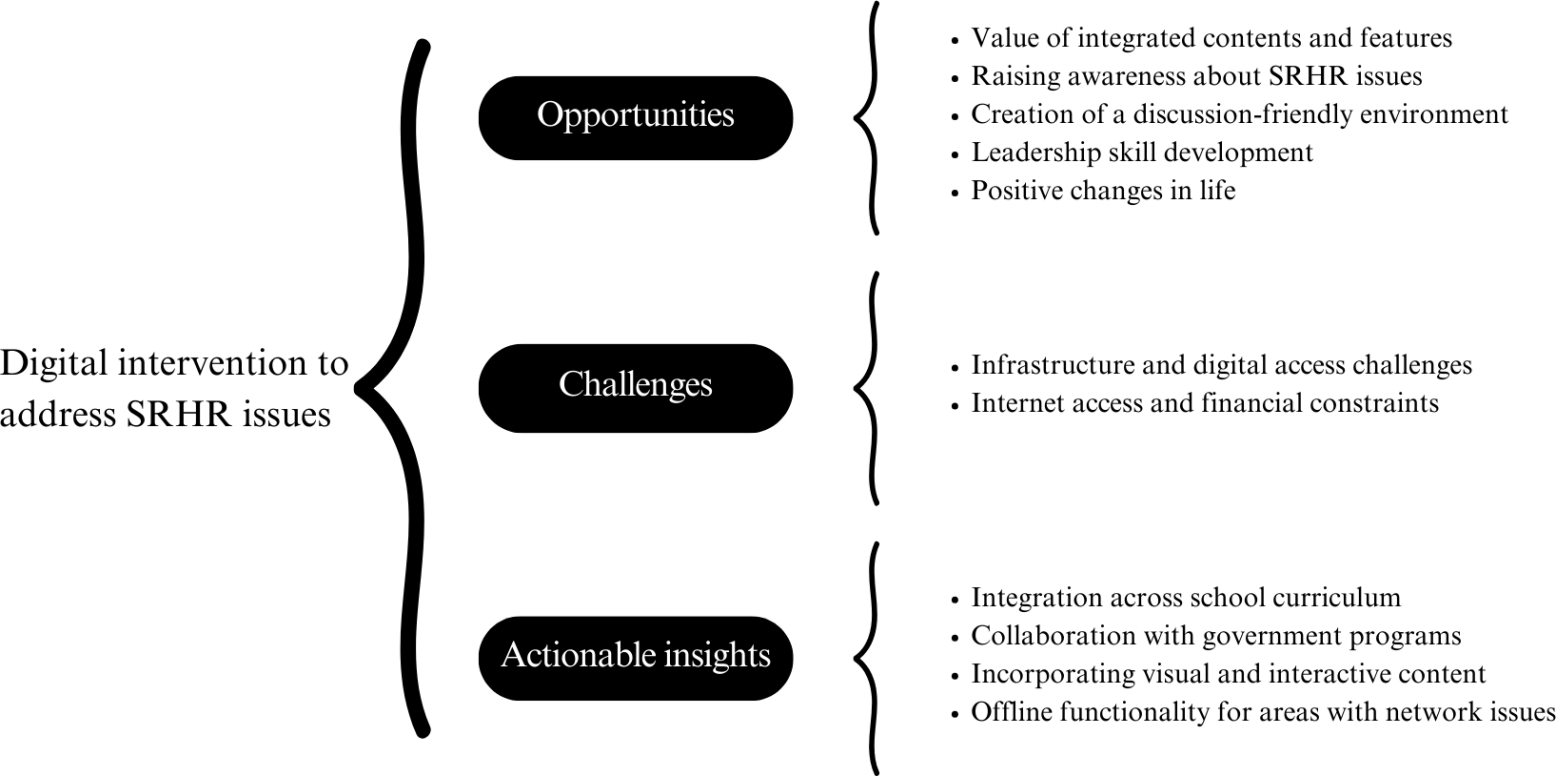
Emerging themes and subthemes during analysis. SRHR: sexual and reproductive health and rights.

All the interviews were audio-recorded and stored in a secure cloud space and were only accessible to the core research team. The recordings were transcribed verbatim in Bengali by experienced transcriptionists who were familiar with qualitative research and subsequently translated into English. To maintain translation accuracy, it was reviewed by the research team, and discrepancies were resolved by consensus. To ensure confidentiality, participants’ names were anonymized during transcription, and each participant was assigned a unique identification number. Inductive thematic analysis was performed [23]. This process was undertaken in six stages: (1) becoming familiar with the transcribed data through repeated readings; (2) generating initial codes and gathering data under each code; (3) developing themes and subthemes; (4) reviewing themes and formatting a thematic matrix for further analysis; (5) defining and naming themes; and (6) writing up. These steps were followed to create a codebook (Figure 2) and write the interpretation. Coding was carried out by the first author (KH) and a research assistant using NVivo software (QSR International) [24]. During the analysis process, researchers used a methodical approach that included regular meetings to review and discuss the coded data [25,26]. Instances of both consensus and disagreement were carefully analyzed and resolved collaboratively [27,28]. To ensure scientific rigor, triangulation of various data sources, such as different types of participants, using different interview methods (KII, IDI, and FGD) was applied. Subsequently, we checked the manuscript by following the 32-item COREQ (Consolidated Criteria for Reporting Qualitative Research) checklist [29] to ensure transparency in all aspects of our study.

### Ethical Considerations

The research protocol (Protocol no: PR-22124) was approved by the Institutional Review Board (IRB) of icddr,b (previously known as International Center for Diarrhoeal Disease Research, Bangladesh). Informed written consent was obtained from teachers and the GoB program officials. For the participants (students and peer leaders) who were under 18 years, informed written assent from students and peer leaders was obtained, and additional informed written parental consents were obtained from their legal guardians [30]. The consent and assent forms were in both Bengali and English, and generally, the Bengali versions were used during interviews. While obtaining consent and assent, the purpose, procedures, risks, and benefits of the study were explained. The participants were also informed that the data would be accessible only for the research team and they had the right to withdraw or skip the questions during the interview without any repercussions, their names would be anonymized to avoid the risk, and it was voluntary participation.

## Results

### Background Characteristics of the Participants

The majority of respondents in this study were female (11/19, 58%). A significant portion (9/19, 47%) of the participants were between the ages of 10 and 15 years, and 4/19 (21%) participants were between 16 and 19 years, considered as the prime target users of the app, and were at the time of this publication enrolled in the ninth grade in the government school. The GoB program officials who participated in the KIIs were 41 years or above, all of whom had completed a university-level education (Table 1).

**Table 1.** Characteristics of participants (N=19).

Characteristics	Students (n=3)	Peer leaders (n=10)	Teachers (n=3)	GoB^a^ program officials (n=3)	Total participants (N=19)
Sex, n (%)					
Male	1 (33)	3 (30)	1 (33)	3 (100)	8 (42)
Female	2 (67)	7 (70)	2 (67)	0 (0)	11 (58)
Age (years), n (%)					
10‐15	2 (67)	7 (70)	0 (0)	0 (0)	9 (47)
16‐19	1 (33)	3 (30)	0 (0)	0 (0)	4 (21)
20‐30	0 (0)	0 (0)	0 (0)	0 (0)	0 (0)
31‐40	0 (0)	0 (0)	3 (100)	0 (0)	3 (16)
≥41	0 (0)	0 (0)	0 (0)	3 (100)	3 (16)
Year of schooling, n (%)					
6‐10	3 (100)	10 (100)	0 (0)	0 (0)	13 (68)
≥11	0 (0)	0 (0)	3 (100)	3 (100)	6 (32)

a GoB: Government of Bangladesh

### Opportunities of the Mukhorito App

The findings revealed several key opportunities associated with the Mukhorito app in addressing adolescent SRH needs in Bangladesh. These opportunities underscored the app’s potential to enhance knowledge, foster leadership, and create a more open and supportive environment for discussing sensitive topics.

#### Value of Integrated Contents and Features

The Mukhorito app was widely appreciated by all users for its structured, easy-to-navigate content, which allowed adolescents to access information efficiently. A participant from ninth grade emphasized the clarity of the app’s design:

*The Mukhorito app organized everything into eight sections. When I want to know something specific, I know where to find it very easily.*

[Peer leader, female, 15 y, P12]

This well-organized structure facilitated seamless access to SRH information, ensuring that users could locate specific content without confusion. The intuitive navigation enhanced the user experience, making the app a practical and user-friendly educational tool for adolescents.

In addition to its structure, the app incorporated engaging elements, such as stories that follow each topic. These narrative features made the learning process more relatable and engaging for students, helping to reinforce key SRH messages in a way that resonated with their daily experiences. An FGD participant highlighted the importance of these stories:

*One thing I like about the Mukhorito app is the stories provided after each topic. […] the story explained the topic in an interesting way.*

[Peer leader, male, 16 y, P10]

According to the participants, the integration of stories helped contextualize educational content, making it both engaging and relatable for users. By weaving SRH topics into compelling narratives, the app not only delivered critical information but also fostered a personal connection, enhancing users’ understanding and retention of the subject matter. In addition, the acceptance of the app’s structured design and inclusion of narrative content revealed significant opportunities to improve adolescent SRH education. These features made the app a valuable tool for adolescents seeking accessible, engaging, and effective learning resources on SRH, further contributing to its role in promoting informed and empowered youth.

#### Raising Awareness About SRH Issues

The Mukhorito app effectively raised awareness about SRH among adolescents. It provided access to essential information on topics such as reproductive health, nutrition, early marriage, and drug addiction that were previously less accessible through traditional educational methods. A student expressed her satisfaction with the app’s content:

*I am satisfied with the Mukhorito app. By using it, we can access various types of information that we didn't know before, such as reproductive health issues and drug addiction.*

[Student, female, 15 y, P2]

Furthermore, students and teachers opined that the app addressed gaps in adolescents’ understanding of nutrition and behavior during puberty through a comprehensive approach. It provided information on adolescent nutrition, puberty-related challenges, and coping mechanisms. For instance, it offered guidance on nutritious foods to combat anemia. A student noted how the app improved his knowledge of healthy eating during adolescence:

*Previously, I didn’t know what better to eat or do during adolescence. Now, I can learn about these things from the Mukhorito app… Because we got the nutrition-related detailed information which is organized in the app.*

[Student, male, 16 y, P03]

A teacher shared that:

*Through this app, they [students] are becoming aware of adolescent nutrition, the issues that affect them during puberty, and how to cope with those effects. Additionally, they are learning about nutritious foods and what to eat during this time to address anemia.*

[Teacher, female, 38 y, P05]

By providing timely and relevant information, the Mukhorito app equipped students with essential knowledge on physical, mental, and reproductive health. This early education prepares adolescents to manage their well-being more effectively, starkly contrasting with previous generations who often acquired such health knowledge later in life. A KII participant pointed out:

*They [adolescents] had no idea about physical health, mental health, or reproductive health. Now, children from those schools are very aware of sexual and reproductive health and mental health. There have been many changes in them. They should know these things at this age. When are we supposed to know the things that we know at this age? Knowing these at the age of 40-50 is useless.*

[GoB program official, male, 53 y, P08]

The Mukhorito app made significant strides in enhancing adolescents’ awareness of SRH issues. The app fostered a more informed and health-conscious generation by providing essential knowledge on reproductive health, nutrition, and mental health. This app played a crucial role in empowering adolescents to understand and address health concerns, both for themselves and their peers. It facilitated a ripple effect of learning that extended beyond individual users, promoting health awareness within families and communities. A female participant illustrated how the app’s knowledge enabled her to educate others, such as her younger sister, about essential aspects of adolescent health:

*I have learned. I will be able to inform someone else. Like, my sister is gradually growing up. I will be able to explain it to her as well. If I understand, then I can inform and explain to others about adolescent health.*

[Student, female, 14 y, P01]

This capability to share knowledge underscored the app’s role in fostering a culture of health awareness among adolescents. It empowered users to take responsibility for their own well-being and to assist others in their journey through adolescence, thereby enhancing community health literacy.

#### Creation of a Discussion-Friendly Environment

The introduction of the Mukhorito app contributed to creating a more open and discussion-friendly environment for adolescents in rural Bangladesh, particularly in the context of SRH topics. Previously, students often refrained from discussing SRH-related issues with teachers or even within their peer groups due to cultural taboos and a lack of appropriate platforms. The app enabled students to engage in conversations about topics by providing a chatbox and facilitating online sessions on subjects that were once hidden or considered inappropriate for discussion. As a result, students could openly discuss their concerns with their peers. According to a teacher, the app allowed previously suppressed concerns to surface:

*This is a very good approach. Previously, they used to hide many things and couldn’t share them with guardians, teachers, or elder siblings. Now, through using this app, issues they couldn't previously share have come to light. They are learning a lot more. There was no way to know these things effectively before. Because of using the app, they are becoming aware and solving problems through increased awareness among themselves.*

[Teacher, male, 37 y, P07]

Thus, the app improved awareness, enabling students to not only discuss SRH issues more freely but also to solve related challenges collectively through peer support and increased self-awareness. This transformation reflected a substantial shift in the learning environment, where social taboos around SRH were progressively dismantled, fostering an atmosphere of open communication and mutual learning.

The findings also highlighted a perceived gradual decrease in the hesitation among adolescents to engage in discussions related to SRH. Although the progress was not fully realized, students began to overcome their reluctance to talk about SRH issues, a hesitation deeply rooted in cultural norms and taboos. A participant shared that:

*From what I have observed, there hasn't been a lot of change yet. But they are now discussing some things with each other. They were not used to discussing SRH issues because there was hesitation among them. That hesitation has lessened somewhat, but complete progress will hopefully happen later.*

[Teacher, female, 35 y, P04]

While the level of openness remained incomplete, there was a clear indication that students were becoming more comfortable discussing these topics, particularly within peer groups. The gradual reduction in hesitation points to the potential for further advancements in SRH education, provided that continued support and sustained engagement are maintained to address the underlying cultural barriers. This trend suggests that more comprehensive progress in open communication regarding SRH matters can be expected.

#### Leadership Skill Development

The Mukhorito app not only served as a platform for adolescent health education but also played a pivotal role in nurturing leadership skills among adolescents so that they could discuss SRH issues and decision-making with confidence. Through engaging in online and interactive sessions, the app helped develop key communication and leadership abilities such as decision-making, initiative, and responsibility. According to the participants, the online sessions allowed them to ask questions when they did not understand or disagree with any discussed topics, even sensitive SRH issues, which increased their communication and cognitive ability. This early exposure to leadership practices equipped adolescents with skills that will benefit them in future professional endeavors, such as job interviews or other challenging environments. By offering task-oriented sessions that required active participation, the app empowered users to build confidence in their responsibilities and roles. A participant stated that:

*Many of us have ambitions for the future. When we go for job interviews or want to achieve something, we are often asked to do different tasks. Through these sessions, we learn leadership skills from an early age.*

[Peer leader, female, 14 y, P13]

#### Positive Changes in Life

The Mukhorito app led to noticeable positive changes in the lives of adolescents, particularly in terms of their attitudes and behaviors toward SRH topics. Several respondents highlighted the app’s role in helping students overcome negative emotions like fear, shame, and embarrassment, which had previously inhibited open discussions. A teacher shared her observation that students who once endured in silence are now empowered not only to address their own SRH issues but also to raise awareness among their peers:

*Yes, since the app was launched, they have overcome various negative effects. For example, they used to keep these problems to themselves and endured in silence. Now, they are not only aware of these issues but also able to raise awareness among others. They can discuss their problems openly, and the doubts or shame that caused fear are no longer present.*

[Teacher, female, 38 y, P05]

In addition, the increased awareness facilitated by the app shaped how students perceived and responded to SRH topics. What was once met with discomfort or mockery was approached with understanding and acceptance during the app’s use. A participant noted a significant behavioral shift in the classroom:

*Students who used to joke around during classes about adolescent issues, now due to increased awareness, learned to accept these issues more easily. This change is evident among them.*

[Teacher, male, 37 y, P06]

Echoing this sentiment, a female peer leader shared her experience regarding a reduction in embarrassment among students, particularly during lessons on physical and sexual education:

*In the past, when we read a topic or chapter on physical education, they would laugh at anything that was said. They thought it was very embarrassing and shouldn't be discussed openly. But now, it has decreased significantly. No one laughs anymore. They take these topics normally*.

[Peer leader, female, 15 y, P17]

By reducing stigma and creating a platform for informed discussions, the app contributed to positive behavioral and attitudinal changes in students’ lives.

### Key Challenges of the Mukhorito App

Despite the positive impact of the Mukhorito app on adolescent SRH education, several challenges hindered its widespread adoption, particularly in rural areas. These obstacles can be broadly categorized into technical and financial constraints.

#### Infrastructure and Digital Access Challenges

One of the primary challenges was the limited access to smartphones, particularly in suburban and rural areas. According to participants, guardians often think of mobile phones as a hindrance to textbook-focused study, and even when students had access to mobile phones, poor network connectivity posed another substantial barrier. A teacher highlighted this as a key issue:

*Many students in predominantly rural areas do not have smartphones. This is the main issue which causes many students to face difficulties in using the app. They say, “Sir, we don't have smartphones, so we can't use the Mukhorito app.” Even if they have smartphones, the network is still not readily available in many rural areas. This makes it difficult for them to participate in sessions.*

[Teacher, male, 37 y, P06]

Another significant challenge in accessing was timing, exacerbated by their limited access to personal mobile phones, as many students depend on shared devices in the family, requiring guardian permission for use. Moreover, a substantial portion of their day was spent in school (9 AM-4 PM), and when they returned home, the phone owner—often their father or brother—may have gone away to the market or elsewhere, making the device unavailable. This restriction on mobile phone access, combined with limited free time outside school hours, contributed to delays in engaging with the app and impeded students’ ability to stay on track with the app. A participant noted:

[…] *most of them don’t have personal mobile phones. They might use someone else’s. They spend most of their time outside, with us at school. So, when they go home, they can use a phone only if their guardian allows it. Since they can't use it personally, they lag behind a bit*.

[Teacher, female, 35 y, P04]

These interconnected barriers, such as the lack of personal smartphones, poor network connectivity, and restricted access to shared devices, underscored the complexities of implementing app-based educational programs in suburban and rural contexts.

#### Internet Access and Financial Constraints

Unreliable internet connectivity represented a significant challenge to effectively piloting the Mukhorito app, particularly in rural and remote areas. Students faced persistent challenges, such as poor network availability, the lack of mobile data, power outages, and dysfunctional phones. A participant said that:

*Many of them [app users] mentioned concerns for not attending sessions, such as lack of mobile data, a dysfunctional phone, time constraints, internet problems, power outages, or no Wi-Fi.*

[Peer leader, female, 16 y, P19]

Another barrier identified by participants was the financial burden associated with accessing the internet for app usage. The cost of mobile data and the need for financial resources to sustain continued use of the Mukhorito app were noted as significant concerns. A teacher emphasized the need for financial assistance to alleviate these challenges:

*The issue they face while using the Mukhorito app is financial. When we use the internet, financial support is always needed. In this case, if they continue working with the app, providing them with financial assistance to overcome internet problems would be beneficial.*

[Teacher, female, 38 y, P05]

Addressing these technical and financial barriers will be crucial to ensuring broader access and effective implementation of the Mukhorito app, especially in underserved rural communities.

### Actionable Insights

Based on feedback from various participants, several suggestions emerged to enhance the effectiveness and accessibility of the Mukhorito app, ensuring it can better serve adolescents in Bangladesh.

#### Integration Across School Curriculum

The app should be integrated into the school curriculum for sustainability, and expanding the app’s reach to adolescent students is considered crucial for fostering early SRH awareness. Introducing the app early with age-specific content would allow students to gradually learn about SRH topics as they navigate the different stages of adolescence, building a more informed and resilient generation.

A participant suggested introducing the app from sixth grade onwards:

T*he app should be introduced in all classes of every school, from sixth to tenth grade. This way, sixth-grade students could follow everything right from the start of adolescence*.

[Student, male, 16 y, P03]

#### Collaboration with Government Programs

The Government of Bangladesh has introduced a school-based adolescent health program to enhance students’ physical, mental, and reproductive health. A GoB Program Official suggested aligning the Mukhorito app with government-run adolescent health initiatives to broaden its impact. He stated:

*If the Mukhorito app can be run together with the government’s adolescent health program, then adolescents will be benefited more and learn more about adolescent health. If they can be nurtured with proper health messages at this age, then they will be able to lead the country and the nation later. Healthy people can build a healthy nation. A healthy nation will be developed only if a healthy generation is developed.*

[GoB program official, male, 53 y, P08]

Integrating the app with the government adolescent health program, which shares a similar vision, could ensure a more coordinated and comprehensive approach to adolescent health education, maximizing its reach and effectiveness.

#### Incorporating Visual and Interactive Content

To increase engagement and retention, the inclusion of visually driven, animation, drama-based content was recommended. A respondent emphasized the educational value of incorporating short films or video clips:

*If we can include some film-based or drama-based content, they might learn about the topics while watching the dramas. We tend to learn more from visual and acted content than from reading, as reading often results in less interest or retention. If short dramas or short video clips are included in each content on the app, I think students would be more interested*.

[Teacher, male, 37 y, P06]

Another respondent suggested giving importance to the visualization of the contents by incorporating the animations. He said that:

*The content of the app should be attractive for reading. These aspects need to be made presentable by adding animation. It will make them feel more attracted and it is necessary for sustainability*.

[GoB program official, male, 48 y, P09]

Such visual content could make the learning experience more immersive and relatable, enhancing students’ understanding and engagement with SRH topics.

#### Offline Functionality for Areas with Network Issues

To address the challenge of poor internet connectivity in rural areas, it was suggested that the app could have some offline functionalities. A respondent proposed developing technology that enables app usage without relying on internet access:

*There is a challenge with network problems [mobile network connectivity]. If a technology could be developed to use the app without relying largely on the internet, it would be useful in areas with network disturbance. In other words, if a way could be found to utilize some functions of the app offline, it could still function in areas with network issues.*

[GoB program official, male, 54 y, P07]

Developing offline features and functionalities would ensure that students in remote or underserved areas can benefit from the app, regardless of the strength of internet connectivity, thus expanding its accessibility.

By addressing these recommendations, the Mukhorito can be further enhanced to reach a wider audience, provide more engaging content, and overcome logistical challenges, ultimately contributing to the development of a healthier, more informed generation of adolescents.

## Discussion

### Integration With Prior Studies

This study highlighted positive impacts, needs, and barriers associated with this integrated digital intervention for adolescent SRH services, information, education, and outcomes in LMICs. With its well-structured and user-friendly design, the Mukhorito app has shown promise in increasing adolescent engagement, enhancing knowledge retention, and encouraging open discussions on SRH issues. A previous systematic review emphasizes that mHealth solutions can effectively promote SRH in LMICs [31]. Adolescents, in particular, are more responsive and enthusiastic about using mHealth technologies to overcome barriers to accessing SRH information and services [32-34]. Our study demonstrated the app’s potential to create an engaging and supportive learning environment and service platform regarding adolescent SRH. However, challenges remain, particularly for future scaling, as the usage of apps for health information and services is still not popular here in this context [35].

Several mHealth apps have been piloted to provide health care services in Bangladesh, yet they often overlook adolescent reproductive health issues [36-40]. Moreover, their evaluation results highlighted the critical scope for enhancement and improvement through the design and development process [36-40]. A significant strength of the Mukhorito app is its integration of interactive, narrative-based learning methods, including stories, sessions, chat boxes, and organized content sections, which facilitated ease of use and relatable learning experiences, as revealed from the study findings, aligning with a similar study showing the effectiveness of interactive functions in assisting adolescents seeking SRH support [41].

The findings highlight Mukhorito’s added value in bridging knowledge gaps related to puberty, nutrition, and general SRH topics, issues previously underrepresented in traditional educational approaches and settings [42]. The global evidence supports digital interventions like Mukhorito as accessible and effective tools to deliver health information, especially to underserved communities [43]. Other studies also validate these findings, showing that digital health solutions for SRH are positively received among adolescents in LMICs, providing anonymity and greater accessibility [44-47]. Moreover, the app’s cascading effect, where students share acquired knowledge with peers and family members, suggests a potential broader community impact, similar to outcomes observed in other digital health interventions [48]. With one of its key features, a discussion-friendly environment, the app is poised to gradually overcome cultural taboos that previously hindered open dialogue about SRH [42,49]. This shift signifies a positive cultural movement toward improved sexual health literacy and open communication, aligning with global findings demonstrating how digital platforms help overcome socio-cultural barriers such as stigmatization, discrimination, lack of privacy, and transportation challenges to SRH information and services [50,51]. However, the challenges of the Mukhorito app are consistent with those identified in other digital health initiatives in resource-constrained environments, where restricted smartphone access, poor network and digital infrastructure, and economic barriers persist as challenges to digital health equity and outreach effectiveness [44,45,52,53].

To maximize the Mukhorito app’s impact and address identified barriers, several recommendations emerged from the study findings. Introducing such an app to younger students, beginning in the sixth grade, with age-appropriate content could foster early SRH awareness and support knowledge development gradually through adolescence. Incorporating more interactive and visually engaging content, such as drama-based videos, could further enhance student engagement and retention of SRH knowledge. Developing offline functionality for the app would also address the issue of poor internet connectivity, ensuring accessibility regardless of location. Furthermore, our app incorporated a participatory, peer interaction, gamification elements, user-friendly design, and easy accessibility to the source of SRH information [20,54]. Collaborating with existing government adolescent health programs could further enhance the app’s reach by integrating it into national health initiatives. Thus, existing adolescent SRH apps could integrate evidence-based mHealth engagement techniques and sustainability strategies, such as participatory and user-centered design approaches, to foster a sense of ownership among users, thereby enhancing interface intuitiveness and overall user engagement [55,56].

### Strengths and Limitations

To the best of our knowledge, this is the first qualitative study conducted among adolescents in Bangladesh to explore the opportunities and challenges associated with using an app to raise awareness of sexual and reproductive health. The study generated rich, detailed insights into the app’s impact on adolescents’ SRH awareness and effectively illuminated underlying behavioral and social contexts that may be overlooked in quantitative surveys. However, several limitations should be acknowledged. The findings of this study should not be generalized but carefully considered and contextualized according to the socio-cultural and institutional setting where the study was carried out. The local norms, socio-economic conditions, and the educational environment were some of the factors that influenced the responses of the participants to a great extent, hence limiting the applicability of the findings to broader settings. Nevertheless, these results provide important, contextualized information that could be useful and applicable in similar socio-cultural and institutional settings. Moreover, data saturation in qualitative research can be difficult to achieve, especially in sensitive topics like SRH. This was one of the challenges that we faced in our study because it required a delicate manner of capturing a detailed and rich account of the experiences and views of the participants. It led us to meet the participants multiple times to build rapport before starting the final interviews or FGD. Nevertheless, we managed to overcome these difficulties and compiled rich and detailed information that gave us meaningful insights.

### Conclusion

The Mukhorito app demonstrates a promising approach to improving adolescent SRH awareness in Bangladesh, showing notable benefits in enhancing knowledge, promoting leadership skills, and fostering open communication and SRH service usage. However, for broader scalability and sustained impact, addressing technical, financial, and infrastructural challenges is crucial. By integrating the app into the national school health program, enhancing its multimedia content, and ensuring offline capabilities, Mukhorito can become a pivotal tool in shaping a healthier and more informed generation of adolescents. Future research should consider examining the app’s long-term behavioral impact and adapting its content for diverse age groups or regional contexts to enhance its relevance and reach. Collaboration with government programs will be crucial for implementing these improvements, expanding the app’s accessibility, and ensuring its sustainable influence on adolescent health outcomes in Bangladesh.

## List of Abbreviations

COREQ: Consolidated Criteria for Reporting Qualitative Research
DGHS: directorate general of health services
DSHE: directorate of secondary & higher education
FGD: focused group discussion
GoB: Government of Bangladesh
IDI: in-depth interview
KII: key informant interview
LMIC: low- and middle-income country
mHealth: mobile health
SRH: sexual and reproductive health
UNICEF: United Nations Children’s Fund
